# A New Player in the Hippocampus: A Review on VGLUT3+ Neurons and Their Role in the Regulation of Hippocampal Activity and Behaviour

**DOI:** 10.3390/ijms23020790

**Published:** 2022-01-12

**Authors:** Csilla Lea Fazekas, Adrienn Szabó, Bibiána Török, Krisztina Bánrévi, Pedro Correia, Tiago Chaves, Stéphanie Daumas, Dóra Zelena

**Affiliations:** 1 Institute of Experimental Medicine, 1083 Budapest, Hungary; ghalla195@gmail.com (C.L.F.); szabo.adrienne93@gmail.com (A.S.); torok.bibiana@gmail.com (B.T.); banrevik@gmail.com (K.B.); correiaufpe@gmail.com (P.C.); tiagochaves91@gmail.com (T.C.); 2 Centre for Neuroscience, Szentágothai Research Centre, Institute of Physiology, Medical School, University of Pécs, 7624 Pécs, Hungary; 3 János Szentágothai Doctoral School of Neurosciences, Semmelweis University, 1085 Budapest, Hungary; 4 Neuroscience Paris Seine-Institut de Biologie Paris Seine (NPS-IBPS) INSERM, Sorbonne Université, CNRS, 75005 Paris, France; stephanie.daumas@sorbonne-universite.fr

**Keywords:** vesicular glutamate transporter, hippocampus, sensory processes, learning and memory, emotions, stress, cardiovascular regulation

## Abstract

Glutamate is the most abundant excitatory amino acid in the central nervous system. Neurons using glutamate as a neurotransmitter can be characterised by vesicular glutamate transporters (VGLUTs). Among the three subtypes, VGLUT3 is unique, co-localising with other “classical” neurotransmitters, such as the inhibitory GABA. Glutamate, manipulated by VGLUT3, can modulate the packaging as well as the release of other neurotransmitters and serve as a retrograde signal through its release from the somata and dendrites. Its contribution to sensory processes (including seeing, hearing, and mechanosensation) is well characterised. However, its involvement in learning and memory can only be assumed based on its prominent hippocampal presence. Although VGLUT3-expressing neurons are detectable in the hippocampus, most of the hippocampal VGLUT3 positivity can be found on nerve terminals, presumably coming from the median raphe. This hippocampal glutamatergic network plays a pivotal role in several important processes (e.g., learning and memory, emotions, epilepsy, cardiovascular regulation). Indirect information from anatomical studies and KO mice strains suggests the contribution of local VGLUT3-positive hippocampal neurons as well as afferentations in these events. However, further studies making use of more specific tools (e.g., Cre-mice, opto- and chemogenetics) are needed to confirm these assumptions.

## 1. Introduction

In the central nervous system (CNS), neurons are classified based on the neurotransmitters they express. While Dale’s principle originally stated that one neuron utilises one neurotransmitter, we now know that a cell can express multiple different molecules to communicate [1]. However, even today, it is still regarded such that neurons have one main “classical” neurotransmitter type (e.g., excitatory glutamate (Glu) or inhibitory gamma aminobutyric acid (GABA)) and express numerous other secondary ones, mainly peptides. As these “classical” neurotransmitters are small molecules, they are often intermediates of the metabolism and thus detectable in all cells. Therefore, neuron classification is based mainly on the transporter proteins that pack the neurotransmitters into vesicles, from which the molecules are later released into the synaptic cleft [2]. One of the most abundant types of neurons in the CNS is the glutamatergic cells, which exert excitation in most cases via the release of Glu. Two distinct protein families transport Glu through membranes: the excitatory amino acid transporters (EAATs) and the vesicular glutamate transporters (VGLUTs). EAATs, being responsible for the termination of the synaptic signal, can be found in the plasma membrane of pre- and postsynaptic neurons, as well as in glial cells, and thus cannot be used to characterise glutamatergic neurons [3].

On the contrary, VGLUTs are expressed on neuronal synaptic vesicles’ membrane and thought to be characteristic to neurons only. They belong to the solute carrier family 17, which is a sodium-dependent phosphate transporter family. To maintain balance of charge, pH, and ions, glutamatergic synaptic vesicle membranes contain V-ATPases (proton pumps) as well, which establish acidic pH inside the vesicles. VGLUTs themselves carry not only Glu in its anionic form but also require Cl^−^ and a cation (preferably H^+^ or K^+^) to work [4,5,6]. According to Preobraschenski’s model, in the first conformation state, VGLUTs bind a Glu^−^ and a K^+^ molecule from the lumen, while a Cl^−^ ion is constantly bound due to the high affinity [4]. After changing conformation, in the second state, the transporter lets go of the Glu^−^ and K^+^ inside and instead gains high affinity to Cl^−^ and H^+^, which are transported to the cytosol to restart the cycle.

These glutamatergic synaptic vesicles are diverse, forming one of the building blocks of neuronal heterogeneity [7]. There are three VGLUT isoforms without significant differences in their Glu uptake when tested in in vitro experiments [4,8]. Transgenic animal models provide an opportunity to explore the role of these transporters in vivo. In 3-week-old VGLUT1 knockout (KO) mice, a significant decrease in the slope of field excitatory postsynaptic potentials (fEPSCs) was observed in hippocampal brain slices. In addition, EPSC measured by whole-cell voltage clamp at −70 mV showed significant damage, but in inhibitory postsynaptic currents (IPSCs) at 0 mV, no significant change was detected. Moreover, Purkinje cells in the cerebellum of these animals also showed severe impairment, without alteration in the climbing fibre responses. In addition, the absence of VGLUT1 resulted in a significant decrease in the frequency of mEPSCs, which was thought to be a consequence of the silenced release sites [9].

The VGLUTs show a distinct topological localisation throughout the CNS. Generally speaking, VGLUT1 is mainly present in the cerebral cortex, hippocampus, and cortical areas of the cerebellum [10,11,12,13], while VGLUT2 is more prominent in the subcortical nuclei such as the thalamus, hypothalamus, and different midbrain structures [11,12,14,15] both in human and rodent brain. In certain areas, such as the amygdala, both can be detected. Even then, the two proteins are expressed in separate subnuclei (e.g., VGLUT1 in the lateral and basolateral amygdala, VGLUT2 in central and medial amygdala) or cortical layers (e.g., VGLUT2 is dominant in the cortical layers IV and VI in contrast to other layers) [11,12,13]. Moreover, these two transporters also do not co-localise with other main neurotransmitters, such as serotonin (5-HT), GABA, dopamine (DA), or acetylcholine (ACh) [12].

For many years, VGLUT1 and 2 were the only two glutamatergic markers that were used in neuroscience. However, in 2002, a third isoform, the VGLUT3, was identified [5,16,17]. Since then, numerous studies have been conducted to unravel its role in cell physiology and behaviour and to find an answer as to why it is so unique compared to the other two.

## 2. Characterisation of VGLUT3

### 2.1. Anatomical Distribution of VGLUT3 in the Central Nervous System

The DNA sequence of VGLUT3 is over 70% identical to the other isoforms, and it utilises the same molecular mechanism to load vesicles with Glu [5,6,8,16,17]. Moreover, its presence is enough to induce the glutamatergic phenotype, as Glu release was detected in GABAergic striatal primary cultures infected with VGLUT3-expressing lentivirus. After 14 days, Glu release-induced EPSCs were detected in the infected cells, whereas no activation was observed in the control GABAergic cells [8].

However, VGLUT3 also shows numerous distinctive characteristics. Firstly, its anatomical distribution is unique: while VGLUT1 and 2 show complementary localisation, VGLUT3 appears intermingled with other transporters, appearing mainly, but not exclusively in subcortical structures. On the mRNA level, it has been shown in neurons of the cortex (layers II, III, and VI), caude putamen, amygdala, hippocampus, hypothalamus, nucleus accumbens, habenula, bed nucleus of stria terminalis (BNST), striatum, ventral tegmental area (VTA), substantia nigra pars compacta, and midbrain raphe nuclei [5,6,11,13,16,18,19,20,21,22], with controversial results in the cerebellum (in the granular layer, molecular layer, Purkinje cells reported in [5], but not found by others [11,16]).

Immunohistochemistry on protein level strengthened the mRNA findings: cortical neurons indeed express VGLUT3 alongside the mRNA [20]. Inhibitory interneurons and pyramidal cells expressing VGLUT3 proteins are also present in layers II and III of the cortex as well as boutons, representing VGLUT3+ synapses in layers II, III, V, and VI [23]. In the hippocampus, pyramidal cell bodies and their dendrites are innervated by VGLUT3+ synapses, while the stratum radiatum somas were also VGLUT3-positive [5,6,11,16]. Similar results were shown in the neurons of olfactory bulb, caudoputamen, nucleus accumbens, striatum, hypothalamus, VTA, substantia nigra pars compacta, and raphe nuclei [5,6,13,16,24,25,26,27]. Moreover, VGLUT3 is not exclusively expressed in the nerve terminals or cell bodies but can also be found in dendrites [5,13]. Interestingly, astrocytes [5,28] and ependymal cell [13,16] were also VGLUT3 positive; however, in situ hybridization did not confirm this on the mRNA level [6,19,29].

VGLUT3 is also detectable in the spinal cord. Numerous VGLUT3+ axon terminals can be found in its intermediolateral cell column, where they form both excitatory (asymmetric) and inhibitory (symmetric) synapses, putatively having a role in thermoregulation [22,30,31]. The retrotrapezoid nucleus, responsible for chemoreception, is also innervated by VGLUT3+ projections [32]. However, VGLUT3 mRNA-positive somas were not detected in the spinal cord [31]. Interestingly, in rat, pulpal blood flow was regulated by VGLUT3+ nerve terminals [33], suggesting the possibility of an even more peripheral projection. Moreover, VGLUT3 immunoreactivity was detected in the heart, liver, and kidney but not in intestinal or lung tissue [34]. However, a specific VGLUT3 isoform is characteristic to the CNS.

### 2.2. Glutamate as a Secondary Neurotransmitter in VGLUT3+ Neurons

Another interesting characteristic of the VGLUT3 is the fact it is co-expressed with other molecules that are considered as traditional main neurotransmitters. Controversially, less is known about VGLUT3 co-expression with non-classical, peptide neurotransmitters.

VGLUT3 is often found in symmetric, thus, inhibitory nerve terminals, especially in the hippocampus and the cortex [5,16,20,21,35]. A small portion of cortical GABAergic interneurons that are projecting locally are VGLUT3 positive, and they also co-express neurokinin B and cholecystokinin (CCK) markers. These neurons form basket-like arborisations around other, putatively neurokinin B positive interneurons [20]. In the hippocampus, glutamate decarboxylase positive (GAD+), GABAergic neurons also express VGLUT3, indicating that inhibitory interneurons also release Glu [5,35,36,37].

Around ≈7% of the GABAergic neurons in the BNST are positive for VGLUT3 mRNA, and part of them project to the VTA [21,38]. In the basal nucleus of the amygdala, a subset of CCK+ GABAergic interneurons also express VGLUT3, along with cannabinoid receptor type 1 (CB_1_R) in their axon terminals [27,39]. Interestingly, these neurons show little electrophysiological and no morphological differences compared to their calbindin positive counterparts [27], but they form an interesting invagination type of synapse into the cell bodies of pyramidal neurons [39].

In the striatum, virtually all cholinergic cells co-express the vesicular acetylcholine transporter (VAChT) and VGLUT3 [5,6,16]. In the basal forebrain (horizontal diagonal band of Broca), cholinergic neurons also co-express VGLUT3, however, in a more restricted way [18,35,40,41]. Some of these cells project to the internal plexiform layer of the main olfactory bulb, although electrophysiological measurements showed that postsynaptic currents are derived from nicotinic and GABAergic activation rather than glutamatergic [40]. Other cells from the basal forebrain project to the basolateral amygdala and express both choline acetyltransferase (ChAT) and VGLUT3 [41]. Interestingly, in the amygdala, some CCK and CB_1_R-positive interneurons also express VGLUT3 [27]. In the striatum, VGLUT3 plays a crucial role in the vesicular loading of ACh [42,43] and excites local fast-spiking interneurons via both α-amino-3-hydroxy-5-methyl-4-isoxazolepropionic acid (AMPA) and N-methyl-D-aspartate (NMDA) (both are ionotropic glutamatergic) receptors. It is thought that this co-release of Glu and ACh plays a role in the regulation of locomotor activity [44]. Similar results were found in basal forebrain nuclei such as the medial septum, diagonal bands, and nucleus basalis [18].

Midbrain raphe nuclei are mostly known for their 5-HT content, which is marked by serotonin transporters (SERT). Interestingly, in these cell groups, SERT+ and VGLUT3+ markers are often co-expressed, but they can also be found separately [5,16,19,20,45,46,47,48,49,50]. Terminals originating from serotonergic neurons often co-express VGLUT3 in the cortex, especially in layers II/III [20]. The source of these terminals is mainly in the dorsal raphe (DR) [19,47,49,50,51,52], which also projects to the striatum [49]. Interestingly, these axons form varicosities that are morphologically larger than others [49]. Other projections in the VTA and nucleus accumbens play a role in reward signalling [52,53,54]. In VTA, both 5-HT and Glu originating from VGLUT3+ axon terminals are released, contributing to social stress susceptibility: their inhibition facilitated social avoidance after subthreshold social defeat stress [53]. However, it is unknown whether the VGLUT3+ subpopulation plays a role in this. In another study, it was shown that the VGLUT3+/5-HT+ DR projections to VTA dopaminergic neurons were excitatory and induced DA release in the nucleus accumbens, positively driving conditioned place preference [54]. Similarly, 5-HT and VGLUT3 co-localisation was detected in the DR-basal amygdala projections, possibly contributing to fear memory [55]. In the basal amygdala, the axon terminals either release 5-HT or Glu based on the frequency of firing [56]. Other than these areas, DR VGLUT3+ neurons also project to the substantia nigra pars compacta, different thalamic and hypothalamic nuclei, where they do not necessarily co-express 5-HT, and their somatas are mainly located in the shell region of the DR [19]. Additionally, there is a subset of VGLUT3+ cells in the superior colliculus that also project to substantia nigra pars compacta and form asymmetric and thus excitatory synapses on local dopaminergic neurons [57].

In another known serotonergic nucleus, the median raphe (MR), Glu released from VGLUT3+ vesicles can be the main neurotransmitter, but it can also be found in serotonergic as well as—in small percentage—in GABAergic neurons [19,23,58]. Interestingly, primary raphe cell cultures from VGLUT3 KO mice show vulnerability and are less likely to survive in vitro compared to cells isolated from wild-type animals [46]. However, there seems to be topological heterogeneity in the neurochemical characteristics of differently projecting serotonergic and VGLUT3+ axon varicosities. For example, in the cortex, hippocampus, nucleus accumbens, and striatum, most varicosities expressed both SERT and VGLUT3 markers [49,59]. On the other hand, Voisin et al. [46] showed the opposite results: in the septum, striatum, and hippocampus, these two markers were barely co-expressed in the same varicosities. Similarly, in the hippocampus, second rhombomere (R2)-derived, Pet1+ (transcription factor known to represent serotonergic cells [60]) boutons were mostly VGLUT3+ but not 5-HT+ [61]. However, serotonergic neurons originating from other rhombomeres co-expressed VGLUT3+ and 5-HT in their terminals. As of now, it is unknown whether this is a technical difference (antibody, different animal strains) or physiologically important observation related to functionality. It is important to note that while this segregation (i.e., 5-HT+, GLUT3+, or co-expressed) in MR-hippocampus projections was confirmed [62], it was also highlighted that VGLUT3 may be co-expressed in vesicular monoamine transporter 2 positive (VMAT2+) and 5-HT+ terminals even if they were negative for the SERT marker. As of now, it is believed that this co-expression facilitates the vesicular filling of the main neurotransmitter (so-called vesicular synergy) [42,62,63]. However, in the case of GABAergic co-expression, both pro [64] and contra [65,66] arguments have been published, leaving the question open. A most probable explanation is that the same projection may have different subtypes based upon their co-expression profile, and different authors found different populations in their samples by chance. The co-expression of “classical” neurotransmitters may be further coloured by an array of peptide co-transmitters [67,68].

Similar to the midbrain raphe nuclei, VGLUT3 can be found alone or co-expressed in a subset of putatively GABAergic and/or aminergic cells in the medullary raphe nuclei, such as the raphe pallidus, raphe magnus, raphe obscurus, and parapyramidal area. They send projections to the spinal cord (see earlier) [22,30]. It has also been suggested that VGLUT3 is also co-expressed with VGLUT1 and 2, but this seems to be brain area [17] and species specific (might be different even between rats and mice) [17,69].

Interestingly, VGLUT3 has the ability to signal retrogradely [5,70]. Crepel et al. showed that cerebellar principle cells utilise Glu released from VGLUT3 containing vesicles to retrogradely signal and regulate incoming signals. In the cortex, VGLUT3 is also present in the dendrites of layer II principal cells and may negatively control the input from local interneurons [24].

### 2.3. Electrophysiological Characteristics of VGLUT3

Lentiviruses containing the sequences of the three VGLUT isoforms were used in primary autopathic cultures from VGLUT1 KO hippocampal and VGLUT2 KO thalamic tissue for direct comparison. All three types of VGLUT expression rescued the deficit in EPSC peaks and charges and showed no significant differences from hippocampal VGLUT1 wild-type (WT) neurons or from each other. Thus, it was concluded that all 3 isoforms perform the basic function in a similar manner. However, compared to WT VGLUT1+ and lentiviral-rescued VGLUT1 cells, VGLUT2 and VGLUT3-expressing neurons showed significantly greater release probability indicated by increased paired-pulse depression [8].

Table 1 shows some representative VGLUT3+ neuron populations in comparison to general GABAergic interneurons and their electrophysiological characteristics. Even though they are located anatomically differently, their major characteristics do not vary in great length.

## 3. Implications of VGLUT3 in Physiology

As a recently discovered protein, the exact role of VGLUT3 is not completely understood. Scarce information are available both from the CNS and the periphery.

First of all, VGLUT3 is highly implicated in sensory processes. In the retina, VGLUT3 plays a crucial role in the physiology of a new subtype of putatively excitatory amacrine cells [5,16,34,73]. VGLUT3 is also expressed in the inner hair cells and spiral ganglion cells of the cochlea [74,75,76] as well as in the inhibitory sound-localisation pathway [77]. They are needed for auditory coding, as without properly functioning proteins, the stereocilary bundle structure and synaptic organisation was lost [78]. As a result, VGLUT3 KO mice are deaf [76,79,80]. VGLUT3-mediated glutamatergic neurotransmission is also responsible for noise-induced threshold shifts [81]. Lastly, low threshold mechanosensitive cells in the hairy skin that project to the dorsal horn of the spinal cord with C-type fibres also utilise VGLUT3 to signal pleasant touch information [69,82,83]. However, unpleasant sensory information of an electric foot-shock was not influenced by the lack of VGLUT3 in KO mice [84].

The participation of VGLUT3 in endocrine regulation was confirmed by several studies. Firstly, glutamate may influence the stress response at several points [85], and VGLUT3 might be especially involved in catecholaminergic regulation, as around 25% of the chromaffin cells in the adrenal medulla also express VGLUT3 [86]. The expression of the hypothalamic regulator of the so-called stress axis (hypothalamic–pituitary adrenocortical axis, HPA), the corticotropin-releasing hormone (CRH) was increased in VGLUT3 KO mice and the stressor-induced corticosterone (end-hormone of the HPA axis) elevation was higher in them compared to their WT littermates [84,85,87]. However, we might assume that the role of VGLUT3 might be on remote brain areas influencing the perception/interpretation of the stressor rather than a direct effect on the HPA axis as its presence (both in the somas as well as in afferent fibres) was not confirmed so far on its elements. Moreover, an array of stressors decreases the number of VGLUT3+ neurons in the DR, where inputs from the central amygdala might play a significant role [88].

As for the endocrine regulation, VGLUT3 may play an important role in insulin secretion directly in the ß-cells of the Langerhans islets of the pancreas [89].

The parabrachial nucleus contributes to the sympathetic nervous system and cardiac activity. After stimulating cardiac sympathetic afferents, c-Fos—a neuronal marker for activity—and VGLUT3 co-localisation was found here, indicating a role of VGLUT3 in cardiovascular responses [90]. In line with this, ischemia-dependent changes in the expression of VGLUTs have been reported in a focal ischemia model. Ischemia is one of the leading causes of death worldwide [91]. Even though the central role of Glu and its receptors in the pathophysiology of cerebral ischemia and the effect of VGLUTs for excitotoxicity following an ischemia–reperfusion challenge has long been recognised [92,93,94], data are still sparse on this topic. In contrast to the transient increase in VGLUT1 protein levels during the first 3 days of reperfusion, VGLUT2 and 3 was reported to be downregulated in the cerebral cortex and caudoputamen of rats [95].

## 4. Characteristics of the VGLUT3 KO Mice

Among the three subtypes, VGLUT1 and 2 seem to be utmost important as their lack is fatal: VGLUT1 KO mice die around weaning, while VGLUT2 KO mice die at birth [96,97,98,99].

VGLUT1 KO animals have progressive neurological symptoms, including blindness and incoordination, supporting its role from an early stage of development [100]. At birth, VGLUT1 KO and WT animals are indistinguishable. However, after birth, VGLUT1 expression increases, accounting for 50% of Glu transmission in 3–5-day-old wild-type mice. In VGLUT1 KO animals during the following 2 weeks, a sharp decrease in residual activity was detected, and in mice older than 2 months, the excitatory transmission was essentially eliminated, leading to death.

VGLUT2 KO mice die due to a complete loss of stable autonomous respiratory rhythm, which is generated by the pre-Bötzinger complex [101]. Similar to VGLUT1 KO, the heterozygous VGLUT2 +/− mice are not obviously different from their WT littermates, despite expressing 50% less VGLUT2 protein [99]. However, various behavioural tests presented well that the partial loss of VGLUT2 significantly affected thalamic function. Among other things, conditioned taste aversion and defensive marble burying were impaired, while motor function, learning and memory, acute nociception, and inflammatory pain remained intact. The same study reported a nearly 50% reduction in the amplitude of spontaneous release events in VGLUT1 null hippocampal and VGLUT2 null thalamic cell cultures [99].

Contrary to the other two isoforms, both heterozygous and homozygous VGLUT3 KO mice are viable and reach adulthood without any need for intervention [85]. It is logical to assume that the elevation of VGLUT1 and 2 compensate the absence of VGLUT3 in the KO mice; however, no VGLUT1 and 2 alterations (both at the mRNA and protein level) were found in their brain [42]. Moreover, in adult VGLUT3 KO mice, the expression of other biochemical markers related to cholinergic, dopaminergic, GABAergic, or neuropeptidergic (substance P, encephalin) regulation were equal to the WT.

VGLUT3 KO mice show no major macroscopic anatomical discrepancies in the brain compared to their WT littermates [25]. Although in vitro raphe primary cell cultures that lack VGLUT3 are less likely to survive [46], in vivo VGLUT3 KO mice do not show reduced serotonergic cell number in their midbrain raphe nuclei. However, in the striatum and dorsal hippocampus of KO mice, the number of serotonergic varicosities was decreased, while in the ventral hippocampus, it was increased [46]. On a molecular level, VGLUT3 KO mice show decreased 5-HT [25,62] and ACh turnover [42].

Since the main VGLUT isoform expressed in the striatum, an area that has an important role in the regulation of movement, is VGLUT3, locomotor alteration in VGLUT3 KO mice could be supposed. However, their motor coordination is normal [85]. Interestingly, during short observations (e.g., 5–10 min open field), a reduced locomotion was detectable [84,85], while during more prolonged observations (up to 5 h), even a hyperlocomotion was observed [42], especially during the dark, active phase [102]. The reduced locomotion seems to be due to enhanced anxiety [62,84], leading to a more cautious behaviour in a new environment, while the hyperlocomotion was connected to their altered DA levels [42,102], suggesting its possible role in Parkinson disease.

In line with an altered HPA axis reactivity mentioned earlier [84,85], VGLUT3 mice were repeatedly reported to be more anxious [62,84,87,103]. This anxiety is innate and can be detected already during the early postnatal period (in 8-day-old mice) by enhanced maternal separation-induced ultrasound vocalisation (at 40–90 kHz) [62,87]. Furthermore, increased anxiety-like behaviour is still detectable in adult mice on numerous behavioural tests such as the elevated plus maze [84,103], or in marble burying, and novelty suppressed feeding paradigms [62].

In line with the detected hyperlocomotion, sensitivity to pharmacological treatments was also altered in the VGLUT3 KO mice [42,102,103,104,105]. Cocaine-induced locomotor activity was exaggerated in them [42,103], and their L-DOPA-induced dyskinesia was reduced [102,105]. Additionally, amphetamine-induced locomotion was also decreased after complete deletion of the VGLUT3 gene [106].

In relation to the above-mentioned drug-induced locomotor discrepancies, addictive behaviour was also altered in VGLUT3 KO mice. These animals proved to be more sensitive, since they responded to smaller amount of cocaine in a conditioned place preference test than their WT littermates [107] and were more responsive when it was used as a reward [104]. This might have a human relevance, as variations in the VGLUT3 gene in patients also correlated with severe addiction [104].

The previously mentioned contribution of VGLUT3 to hearing was confirmed by the loss of auditory brainstem responses in VGLUT3 KO animals [76,79,80]. Interestingly, the p.A211V point mutation of VGLUT3 also results in progressive deafness in humans [76]. However, in mice, aside from the progressive deafness, no major behavioural phenotype was observed due to the same point mutation; only an in vitro decreased VGLUT3 expression was detected [108].

As numerous brainstem areas involved in respiration and thermogenesis also contain VGLUT3+ neurons (see earlier), we might assume alteration in these systems as well. Indeed, despite preserved structure, the respiratory rhythm generator neurons of the brainstem in VGLUT3 KO mice fired with decreased amplitude and duration in response to hypoxic stress, which was probably due to an altered 5-HT turnover [25]. Moreover, their thermoregulation was also disrupted [25].

Learning and memory formation in VGLUT3 KO mice was also investigated, and no major disruption was found [109]. Indeed, an earlier study suggested the role of VGLUT1 and 2 rather than 3 in the measured parameters [110]. However, further studies might be needed to reveal the role of local VGLUT3 positive neurons and terminals in the processes leading to spatial, emotional, and other types of memories.

These observed changes are crucial in behavioural neuroscience, as differences between WT and KO mice in the above-mentioned parameters (e.g., locomotion, hearing) might significantly distort other results, such as anxiety-like behaviour, depressive-like behaviour, or learning paradigms.

## 5. The Hippocampus

In mammals, the hippocampus is known to be one of the centres of memory formation, representing the spatial aspects of the context in which they occur [111]. With the help of special neuropeptides (e.g., oxytocin), even socially meaningful territorial memories can be stored [112]. In the process of learning and memory, the role of hippocampal Glu is indispensable [113,114]. However, as part of the limbic system, the hippocampus is deeply implicated in mood regulation as well [115,116]. In emotion-related disorders—among others—an imbalance between Glu and GABA can be found in the hippocampus. As previously said, the hippocampus is rich in both locally produced and afferent VGLUT3 positivity [35,117]; therefore, we can assume its local regulatory role in the above-mentioned (learning and memory as well as emotional) processes.

### 5.1. Characteristics of the VGLUT3+ Neurons in the Hippocampus

**Figure 1:**
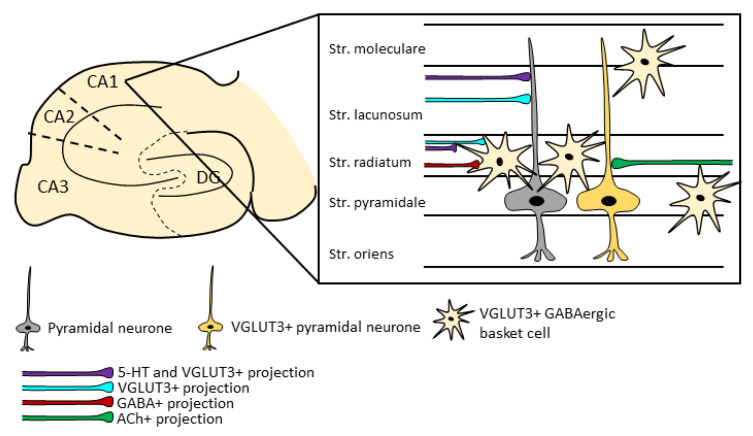
VGLUT3+ neurons and axons in the CA1 region of hippocampus. Local GABAergic interneurons express VGLUT3 and contribute to the proper functionality of the hippocampus. These cells are most prominent in the stratum radiatum, but scarce VGLUT3 positivity can be found in the interneurons of stratum moleculare, stratum lacunosum, stratum pyramidale, and stratum oriens. Additionally, there are some VGLUT3-expressing pyramidal neurons as well. Punctate labelling from in situ hybridisation and immunohistochemistry experiments indicated VGLUT3+ afferents as well. These are mainly localised in the stratum radiatum and often but not exclusively co-expressed with serotonergic markers. The figure was done based on the work of Fasano et al., 2017; Szőnyi et al., 2016; Somogyi et al., 2004; Herzog et al., 2004. 5-HT: serotonin; ACh: acetylcholine; CA: cornu ammonis; DG: dentate gyrus; str: stratum; VGLUT3: vesicular glutamate transporter type 3.

The locally VGLUT3-producing neurons were found to be inhibitory, GABAergic interneurons in the stratum radiatum of the CA1 and CA3 regions, with sparse appearances in the stratum pyramidale, stratum oriens, stratum lacunosum-moleculare, and stratum lucidum. In the dentate gyrus, they appeared in the subgranular zone and few cells in the hilus (Figure 1) [23,35,118]. These were also positive for CCK, ErbB4, and CB_1_R, and very rarely co-expressed calbindin, neuropeptide Y, and somatostatin [20,23,36,65,119]. A double fluorescent in situ hybridisation (FISH) labelling technique was used to detect the GAD-expressing GABAergic neuron population and the proportion of VGLUT3 and vesicular inhibitory amino acid transporter (VIAAT) co-positive cells. It was found that almost all VGLUT3+ cells expressed VIAAT, but only around 9% of VIAAT-positive neurons also contained VGLUT3 in the CA1 pyramidal layer, forming mainly inhibitory synapses [64]. Thus, it is in contrast to the previously mentioned unclear VGLUT3 co-localisation with serotonergic markers.

Based on their morphology, VGLUT3-positive interneurons are rather heterogeneous [65], targeting mainly the pyramidal cells of the hippocampus, but also innervating some interneurons and granular cells [23,35,64,65]. The role of these interneurons is predominantly inhibitory; however, among specific conditions, the excitatory glutamatergic effect might dominate via ionotropic receptors [35,65].

It is known from anatomical and pharmacological studies that metabotropic Glu receptors (mGLUR) 3, 4, 7, and 8 are expressed at the GABAergic terminals in the hippocampus [120]. However, according to Fasano et al. [64], only the presynaptically expressed mGLUR4 may provide inhibitory feedback, putatively activated by Glu released from VGLUT3+ vesicles, and thus, decrease GABAergic output. In support, a strong increase in GABAergic transmission, in the frequency and amplitude of IPSCs, was observed in the absence of VGLUT3. These alterations may also contribute to the observed behavioural changes (e.g., enhanced anxiety) in VGLUT3 KO mice [62].

### 5.2. Hippocampal VGLUT3+ Projections

The hippocampus is rich in afferents. Certain incoming VGLUT3+ fibres also express GAD and occasionally CCK [23]. They synapse on local calbindin+ interneurons or on pyramidal cells. Some of them are showing serotonergic markers as well and could be found in the stratum radiatum and stratum lacunosum-moleculare. Finally, there are also fibres that are not showing any GABAergic, aminergic (characterised by tyrosine hydroxylase negativity), and cholinergic markers.

Many afferents are coming from the brainstem, especially from the raphe nuclei. Although DR also sends axons to the CA1, CA3, and dentate gyrus regions [121], mainly to the ventral hippocampus [122,123], but its main projection targets are in the frontal cortex and striatum [124,125]. On the other hand, MR heavily innervates the hippocampus [126,127], mostly the dorsal part [122,123], through non-serotoninergic, VGLUT3 positive afferents [128]. Indeed, the existence of a functional connection was confirmed by the presence of GluN2A+ (a subunit of NMDA receptor) at local calbindin+ inhibitory interneurons and pyramidal cells in the stratum radiatum, stratum lacunosum-moleculare, stratum pyramidale, granule cell layer, and inner molecular layer [61,117,129,130,131]. Certain axons express either VGLUT3 alone or both VGLUT3 and 5-HT markers [19,23,59,117,132]. Releasing Glu or additionally 5-HT from the VGLUT3+ axon terminals, MR projections may activate hippocampal interneurons, which in turn inhibit local pyramidal cells [132]. These subpopulations may have different in vivo activity [133].

Interestingly, it seems that at least a small portion (≈8–12%) of MR VGLUT3+ cells (and a subpopulation of neurons that are 5-HT+ as well) project to both the hippocampus (CA1, CA3, and dentate gyrus regions) and medial septum at the same time [23,117,126,128]. This raises the question of whether MR synchronises the activity of these brain areas. Indeed, the MR-innervated GABAergic, parvalbumin+ interneurons of the medial septum regulate local cholinergic neurons that act as a pacemaker and control hippocampal theta rhythm and desynchronisation [134,135,136]. Subsequent studies found that the vast majority of these innervations is VGLUT3 positive [117,121,128]. However, it is still unknown whether these MR innervations are responsible for the coordinating role of the medial septal neurons. Nevertheless, there is electrophysiological evidence that MR activation results in the desynchronisation of hippocampal rhythmicity putatively via both direct and indirect medial septal projections [137,138]. There is a bidirectional connection between the hippocampus and MR: the activity of some serotonergic and a significant amount of non-serotonergic cells in the MR was decreased before ripple oscillations and—in turn—right after their synchronous firing, ripple waves were suppressed in the hippocampus, which was also detectable during sleep.

During development, part of the serotonergic neurons express tryptophan hydroxylase 2 (Tph2, important for 5-HT synthesis) in high quantity, while others have high VGLUT3 expression, both showing high Pet1 positivity [61,139]. These Pet1+/VGLUT3+ but 5-HT- neurons project for example to the dorsal hippocampus, forming pericellular baskets [61]. DR neurons show positive correlation between their Tph2 and VGLUT3 mRNA levels [139].

There is also evidence that the VGLUT3+ cells projecting to the hippocampus also send collaterals to the medial prefrontal cortex [117]. This may ensure prefrontal–hippocampal interaction in the process of spatial decision making [140].

### 5.3. Role of the Hippocampal VGLUT3 Positivity

Synchronous activity of the hippocampus is necessary for proper learning and memory formation. Ripple events seem to be essential for spatial memory coding [141,142]. During sharp-wave ripples, the newly acquired hippocampal information is transferred to the neocortex and stored as a long-lasting memory trace (called consolidation) [143,144]. Other hippocampal rhythms, such as theta oscillations, are also characteristic to the hippocampus and are intrinsically created via the subtle interplay between interneurons and pyramidal cells, contributing to learning and memory [145,146]. Theta oscillations are observable in both humans and rodents during spatial navigations [147].

MR regulates hippocampal theta oscillation and thereby memory consolidation [148]. Excitation of the MR impaired the acquisition of fear memory without a negative effect on sleeping. While the authors could not identify the neurochemical characteristics of the cells responsible for it, they showed that serotonergic neurons contribute only to a small extent, while local GABAergic neurons had an indirect role, highly probably via local regulation of the responsible cells. As the vast majority of MR–hippocampal projections are VGLUT3+ (see earlier), thus, we could assume that these projections play a central role in memory formation. Indeed, VGLUT3 and CCK double-positive basket cells seem to be necessary for optimal theta oscillation, offering the basis for normal spatial memory formation (assessed by object location and Morris water maze tests) [36]. Moreover, the deletion of VGLUT3 was shown to induce metaplastic shift to lower the frequency of theta rhythm, indicating an impairment in excitatory–inhibitory balance [64].

However, it has to be mentioned that around 20% of MR neurons are VGLUT2+ [14]. These cells also project to hippocampal parvalbumin+ interneurons, which are also known to regulate theta rhythm and negative memory acquisition.

In relation to emotions, changes in sucrose intake in rodents are known to reflect depressive-like behaviour [149,150]. In this respect, chronic sucrose consumption decreased the number of VGLUT3+ varicosities in the dentate gyrus of the mouse hippocampus independently from their 5-HT marker expression [151]. Interestingly, in rats, repeated sucrose consumption increased VGLUT3 expression in the nucleus accumbens, the centre known for reward, as well [152]. Whether these alterations were due to hippocampal changes remain to be elucidated. However, in an in vitro rat hippocampal neurovascular unit model, glucose and corticosterone-induced depression resulted in an upregulation of VGLUT3 expression [153]. Additionally, the contribution of hippocampal VGLUT3+ neurons to anxiety also needs further clarification. Indeed, previous studies suggested the role of serotonergic input from the MR to the hippocampus in the promotion of anxiogenic behaviour in female mice; however, to what extent the VGLUT3+/5-HT+ subpopulation contributed to this is still unknown [154].

Hippocampal Glu is also implicated in epilepsy. VGLUT3 KO mice showed spontaneous epileptic seizures recorded with electroencephalography; however, these seizures remained behaviourally hidden [79]. Moreover, increased VGLUT3 immunoreactivity—beside reduced VGLUT2 immunoreactivity—was detected in the biopsies of temporal lobe epilepsy patients [155].

In relation to the previously mentioned role of VGLUT3 in cardiovascular regulation, the age-dependent role of hippocampal VGLUT3 was studied to global brain ischemia in 3- and 18-month-old rats [156]. VGLUT3 levels (both at the mRNA (qPCR) and protein (Western blot) levels) were significantly decreased in all hippocampal areas of treated young animals after 48 h of reperfusion. However, VGLUT3 mRNA values were significantly higher in older than in young hippocampi. In contrast to an overall, ischemia-induced decrease, VGLUT3 mRNA levels in CA3 were significantly higher in insulted older animals than in their corresponding controls. Interestingly, an anti-inflammatory drug (the non-steroidal meloxicam) increased the expression of VGLUTs in all animals, ameliorating the outcome of the injury.

## 6. Future Perspectives for Selective Hippocampal VGLUT3+ Manipulations

Based on our current knowledge it is hard to give an exact answer about the role of VGLUT3+ neurons, especially VGLUT3 hippocampal expression in various neuronal subpopulations. As VGLUT3 is expressed in interneurons, its effects can be both local and on a network level. Most of our knowledge is still related to VGLUT3 KO animals, where the possibility of compensation is rather high. At present, we can be sure that VGLUT3 regulates—to at least some degree—anxiety-like behaviour, stress reaction, locomotion, and addiction (see earlier). Whether hippocampal VGLUT3 also participates in these processes remains to be elucidated. Although the learning and memory formation—in which the hippocampus has a pivotal role—of VGLUT3 KO mice also has been studied, only small, fine discrepancies were found [84,109]. Further studies focusing on local, hippocampal-VGLUT3 positive cells as well as projections are needed.

**Figure 2:**
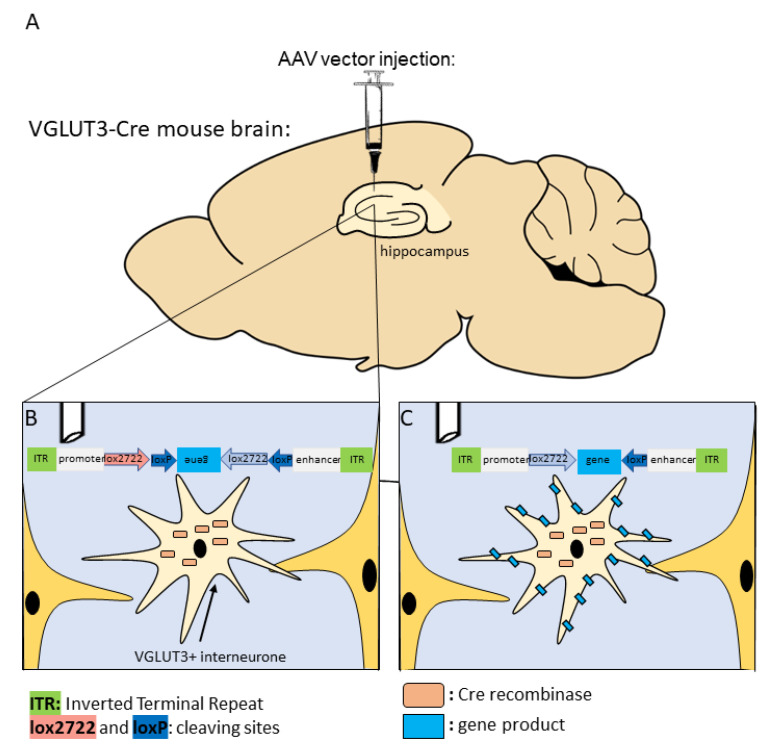
Schematic representation of how VGLUT3-Cre mice work with AAVs. The VGLUT3-Cre mouse strain expresses Cre recombinase enzyme under the promoter of VGLUT3. (**A**) Local microinjection of AAVs—for example, to the hippocampus—can be done via stereotaxical surgery. (**B**) The injected AAVs contain double-floxed inverted open reading frame (DIO) construction. The promoter and enhancer regions enable stable and neuron-specific gene expression. The specific lox-P sites enclose the target gene, which is in the opposite reading order. Thus, non-Cre expressing cells translating these sequences end up with non-functional proteins. (**C**) Cre enzymes in the VGLUT3+ neurons are able to inverse the target gene and translate it into functional proteins. AAVs: adeno-associated viral vectors; VGLUT3: vesicular glutamate transporter type 3.

These specific manipulations became possible with the discovery of the Cre (causing recombinase) enzyme [157]. The Cre enzyme was isolated from the P1 bacteriophage, which can recombine DNA fragments [158]. The binding sites of Cre are the loxP (locus of crossing over/×/P1) sites. Thus, the Cre enzyme recognises the loxP-locus on the target chromosome/gene, which leads to site-specific recombination allowing precise engineering of gene function while maintaining spatial and temporal control of gene expression. Cre-mediated deletion, inversion, or translocation can occur between the two loxP sites (Figure 2). Therefore, gene knocking out, gene activation, or gene transfer can also be achieved with this technique [157]. This tool has easy application and versatility in mammalian genetics and cell biology.

**Figure 3:**
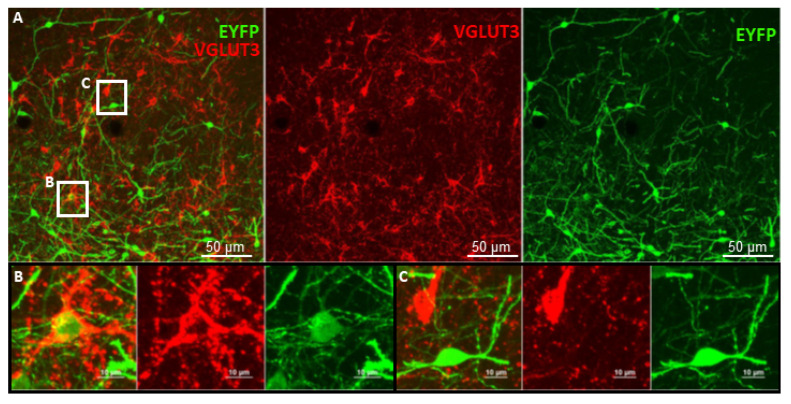
Improper functioning of Cre system in the BNST. (**A**) VGLUT3-Cre mouse acquired from the Jacksons Laboratory (USA; stock No.: #028534) and bred locally at the Institute of Experimental Medicine was injected with AAV containing the sequence of EYFP in a DIO construct (Addgene, catalog No.: #20298-AAV5) (see Figure 2B). Brain slices of 30 μm thickness were stained with a-VGLUT3 (rabbit, Synaptic Systems, stock No.: #135203) and a-EYFP (chicken, Life Technologies, stock No.: #A10262), then with Alexa-594 conjugated a-rabbit (goat host, Life Technologies, stock No.: #A11012) and Alexa-488 conjugated a-chicken (goat host, Life Technologies, stock No.: #A11039) antibodies, respectively. EYFP-positive (green) cells represent Cre activity. (**B**) Example of a proper Cre system functioning: VGLUT3+ (red) neuron was able to inverse and express the gene sequence of EYFP that was coded in the injected AAV in the BNST. (**C**) Example of an improper Cre system functioning: numerous other cells in the BNST that are VGLUT3+ (red) failed to express the EYFP, while VGLUT3 negative neurons were able. Thus, this VGLUT3-Cre mouse could not be used for the specific manipulation of BNST VGLUT3+ neurons. Double fluorescent immunohistochemistry was done by László Bíró, and the pictures were taken by him at the Nikon Centre at the Institute of Experimental Medicine with C2 confocal laser-scanning microscope at 20× and 60× magnification. AAV: adeno-associated viral vectors; BNST: bed nucleus of stria terminalis; DIO: double-floxed inverted open reading frame; EYFP: enhanced yellow fluorescent protein; VGLUT3: vesicular glutamate transporter type 3.

The Cre mice are genetically modified strains that can express the Cre enzyme under specific promoters. Only in these specifically labelled cells, containing the Cre, will the gene of interest flanked by two loxP sites be expressed and inserted to the brain with the help of a vector (mainly adeno-associated viruses [159]) or through cross breeding. Although Cre recombinase transgenic mice are widely used in modern science, researchers still face some drawbacks regarding this model. For instance, it is imperative to examine the expression of loxP-flanked target sequences in a specific cell population of different tissues, even on different brain areas, due to enormous variations in the sensitivity of loxP-flanked target genes to Cre-mediated recombination (Figure 3) [160]. Furthermore, the recombination of inducible Cre systems must be analysed not only before but also after Cre induction [161]. Reports regarding detrimental effects of homozygous Cre transgenic mice are not uncommon [162,163]. Another potential issue of this technique is the so-called Cre toxicity, where Cre recombinase attack cellular DNA, leading to abortive pregnancies [164], through abnormal development of the animal to almost complete obliteration of specific tissues. Homozygous insertion of the Cre enzyme might delete the target gene (e.g., lower DA level in DA transporter Cre mice). Therefore, using heterozygous Cre mice for research purposes is recommended, preferably originating from “normal” mother x homozygous father mating. However, possible parents-of-origin effects should also be taken into consideration. Another important issue is the “ectopic” expression, when Cre appears in non-target cells [165]. A possible explanation of this is that at some point during development, these cells expressed the target genes, which remained Cre-positive even in adulthood.

When Cre mice are mated with another strain containing the target gene between two loxP loci, the breeding must be set up in such a way that the Cre allele and the loxP-flanked allele are inherited from different parents. If this issue is ignored, then some of the animals may carry the desired conditional mutation, whereas others may have a larger fraction, if not all, of the body’s cells mutated [166]. Thus, inconsistent recombination among littermates, as well as mosaicism, may occur.

**Figure 4:**
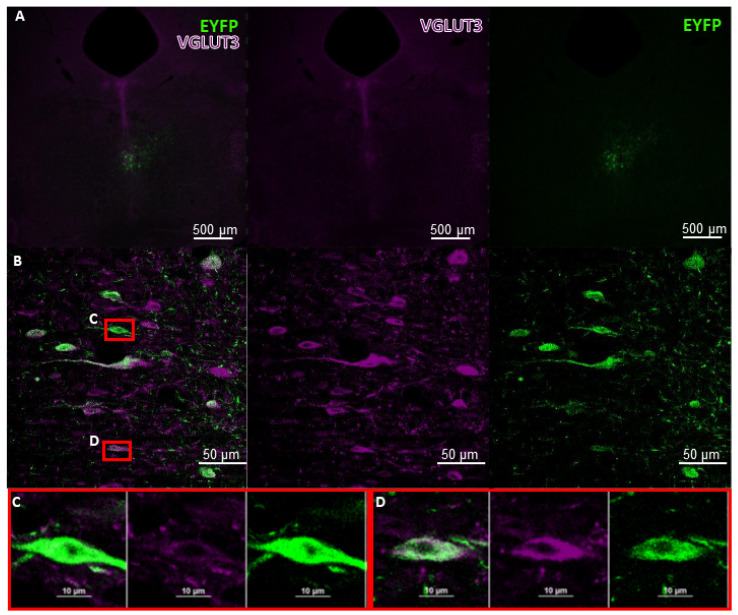
Proper functioning of Cre system in the MR. VGLUT3-Cre mouse acquired from the Jacksons Laboratory (USA; stock No.: #028534) and bred locally at the Institute of Experimental Medicine was injected with AAV containing the sequence of EYFP in a DIO construct (Addgene, catalog No.: #20298-AAV5) (see Figure 2B). Brain slices of 30 μm thickness were stained with a-VGLUT3 (rabbit, Synaptic Systems, stock No.: #135203) and a-EYFP (chicken, Life Technologies, stock No.: #A10262), then with Alexa-647 conjugated a-rabbit (donkey host, Jackson, stock No.: #711-605-152) and Alexa-488 conjugated a-chicken (goat host, Life Technologies, stock No.: #A11039) antibodies, respectively. EYFP-positive (green) cells represent Cre activity. (**A**) Viral staining in the whole MR. (**B**) Example of a proper Cre system functioning: VGLUT3+ (purple) neurons were able to inverse and express the gene sequence of EYFP that was coded in the injected AAV in the MR. We found no ectopic (that is, EYFP in non-VGLUT3+ cells) expression. (**C**,**D**) Double immunofluorescent positive neurons in the MR. Double fluorescent immunohistochemistry was done by László Bíró, and the pictures were taken by him at the Nikon Centre at the Institute of Experimental Medicine with C2 confocal laser-scanning microscope at 4×, 20×, and 60× magnification. AAV: adeno-associated viral vectors; EYFP: enhanced yellow fluorescent protein; DIO: double-floxed inverted open reading frame; MR: median raphe; VGLUT3: vesicular glutamate transporter type 3.

To avoid the above-mentioned problems, deep characterisation of the Cre strains is necessary [167]. Indeed, some VGLUT3-Cre mouse strains are already validated and commercially available. With appropriate viral vectors, they enable the cell-specific expression of any given gene sequence (Figure 4), such as artificial ion channels for optogenetics, or receptors for chemogenetics.

Although optogenetics can be traced back to the 1970s, the modern usage of it was only introduced in the past decade. This technique is famous due to its high spatiotemporal resolution, allowing researchers to turn on or off neuronal activity in a millisecond, similarly to what happens in real life. Microbial opsin genes that code light sensitive ion channels were altered to develop the proteins we use today. They possess faster kinetics and are easier to modify than animal opsins [168]. In neuroscience, the most used opsin is channelrhodopsin-2 (ChR2), originating from the green algae *Chlamydomonas reinhardtii* [169]. This opsin is only activated by blue light, and it causes excitation via cation (Na^+^, H^+^, K^+^, Ca^2+^) influx [170]. Archeorhodopsins and halorhodopsins, both derived from *Halobacteriums*, are activated by green or yellow light, and they hyperpolarise neurons via H^+^ efflux or Cl^−^ influx, respectively [159]. One of the limitations of optogenetics is the invasive surgical implantation of the optic fibres to deliver light to the desired area. However, new enhancement has been done in the past years to minimise the invasiveness of this technique. In fact, recent research by the laboratory of Karl Deisseroth successfully demonstrated deep brain optogenetics stimulation without intracranial surgery [171].

Chemogenetics (or pharmacogenetics) is a technique that enables the remote chemical control of cell populations by using artificially engineered receptors and ligands that are biologically inert. The most common engineered receptors are modified human muscarinic G-protein coupled receptors, such as designer receptors exclusively activated by designer drugs (DREADDS). DREADDS have been used in several animal models to target and affect the activity of numerous cells. Many DREADDS have been developed that can either increase [172] or decrease [173] the neuronal activity. Differently from optogenetics, which requires the invasive insertion of optic fibres, chemogenetics only need the application of the artificial ligand, which can be done via systemic injections, microinfusions, or via the drinking water [174].

The above-mentioned techniques enable researchers to answer very specific questions both in terms of spatial and temporal resolution. In the next few years, more and more studies will be conducted that investigate the role of the separate VGLUT3+ subpopulations within the brain at local, network, and behavioural levels.

## 7. Conclusions

The hippocampal glutamatergic network plays a pivotal role in several important processes (e.g., learning and memory, emotions, epilepsy, cardiovascular regulation), and recently, the involvement of its specific glutamatergic subpopulation characterised by the presence of VGLUT3 was also suggested in these phenomena. Indirect information from anatomical studies and KO mice strains suggests the contribution of local VGLUT3 positive hippocampal neurons as well as projections in these events; however, further studies utilising more specific tools (e.g., Cre-mice, opto-, and chemogenetics) are needed. Nevertheless, VGLUTs could be a target for therapeutical intervention, and already, a variety of antagonists has been developed and tested [175,176,177,178].

## Figures and Tables

**Table 1:** Electrophysiological characteristics of different VGLUT3 containing and non-containing interneurons in the central nervous system.

	GABAergic Interneurons in the Cortex	GABAergic Interneurons in the Hippocampus	VGLUT3+ Interneurons in the Amygdala	VGLUT3+ Interneurons in the Hippocampus
**Resting membrane potential**	−57.48–−49.40 mV	NA	NA	−59.00–−56.90 mV
**Input resistance**	219.77–419.61 MΩ	107.89 MΩ	168.10 MΩ	149.70–158.50 MΩ
**Action potential threshold**	−32.67–−27.82 mV	−42.81 mV	−38.80 mV	−41.90–−39.86 mV
**Action potential amplitude**	71.30–86.11 mV	74.27 mV	71.60 mV	55.70–57.40 mV
**Firing frequency**	19.34–52.48 Hz (2×)	15.00 Hz (steady trace)	31.50 Hz (2×)	31.30–34.90 Hz (2×)
**Amplitude of after-hyperpolarisation**	8.60–17.63 mV	12.68 mV (new method)	14.70 mV	−11.80–−10.30 mV
**Co-transmitters**	CCK	CCK	CCK, GABA	CCK, GABA
**Reference**	[71], all subtypes displayed	[72]	[27]	[65], both subtypes displayed

The expression of VGLUT3 does not change the main properties of the interneurons. For detailed information and results, please refer to each original research article. CCK: cholecystokinin; NA: not available; VGLUT3: vesicular glutamate transporter type 3.

## Data Availability

The review does not contain any new data.
